# Deep learning in ventral hernia imaging: automated multi-structure CT segmentation for surgical planning

**DOI:** 10.3389/jaws.2026.15545

**Published:** 2026-08-07

**Authors:** Vinayak Rengan, Pravin Meenashi Sundaram, Eham Arora, Sabari Girieasen, Ashvind Bawa, Naveen Alexander, Rengan Ravanasamudram Sitaraman, Vimalakar Reddy, Vishakha Kalikar, Aman Arora, Lakshmi Kona, Rochita Venkataramanan, Devansh Lalwani, Dakshin Meenashi Sundaram, Rohit Kalla

**Affiliations:** 1 Department of Pediatric Surgery, Dr. Mehta Hospital, Chennai, India; 2 Department of General Surgery, Sheffield Teaching Hospitals NHS Foundation Trust, Sheffield, United Kingdom; 3 Department of General Surgery, Grant Government Medical College and Sir J J Group of Hospitals, Mumbai, India; 4 Department of General Surgery, Madras Medical College and Government General Hospital, Chennai, India; 5 Department of General Surgery, Dayanand Medical College and Hospital, Ludhiana, India; 6 Department of General Surgery, Sri Ramachandra Medical College and Research Institute, Chennai, India; 7 Department of General Surgery, Chennai Hernia Centre, Chennai, India; 8 Department of GI Surgery, KIMS-Sunshine Hospital, Hyderabad, India; 9 Department of General Surgery, Zen Multi Speciality Hospital, Chembur, India; 10 Department of General Surgery, Army Medical Corps Centre and College, Lucknow, India; 11 Department of GI Surgery, Yashoda Hospital, Hyderabad, India; 12 Department of Radiology, Advantage Imaging and Research Institute, Chennai, India; 13 King Edward Memorial Hospital and Seth Gordhandas Sunderdas Medical College, Mumbai, India; 14 Department of Pediatrics, Saint Peter’s University Hospital, New Brunswick, NJ, United States; 15 Curium Life Tech, Chennai, India

**Keywords:** artificial intelligence, computed tomography, computer vision, hernia imaging, surgical planning

## Abstract

**Background:**

Accurate preoperative assessment of ventral hernia defects remains time-intensive and subject to inter-observer variability. Current manual CT analysis for surgical planning is time-consuming, with inconsistent measurements affecting operative decision-making.

**Methods:**

215 CT scans of adults with ventral hernias were analyzed using TransUNet-inspired deep learning models. Expert annotations of anatomical landmarks and hernia features served as ground truth. Models were trained to automate segmentation of hernia defects and other critical anatomical structures.

**Results:**

Automated segmentation achieved IoU values of 0.85 for hernia defects, 0.89 for rectus abdominis muscles, 0.87 for lateral abdominal wall muscles, and 0.91 for psoas muscles.

**Conclusion:**

Deep learning automation provides rapid, standardized hernia assessment for surgical planning. The system delivers objective measurements with significant time savings, demonstrating technical feasibility as a proof-of-concept that warrants further prospective clinical validation before deployment in operative decision-making.

## Introduction

Contemporary ventral hernia repair demands precise preoperative assessment of defect morphology, abdominal wall anatomy, and patient-specific factors that influence surgical approach and outcomes [1, 2]. The complexity of modern hernia surgery, particularly in cases requiring component separation or complex reconstruction, necessitates detailed imaging analysis that extends far beyond simple defect measurement [3]. Current preoperative CT assessment relies on manual measurement techniques that are both time-intensive and subject to significant inter-observer variation [4]. This variability directly impacts critical surgical decisions including mesh selection, operative approach, and need for component separation techniques, affecting clinical decision-making in up to 56% of cases [5].

The challenge is compounded by the increasing complexity of hernia presentations seen in modern practice. Large defects, loss of domain scenarios, and multiply recurrent hernias require sophisticated analysis of anatomical relationships that traditional measurement approaches struggle to standardize [6]. Furthermore, the growing emphasis on evidence-based hernia surgery demands consistent, reproducible measurements for outcomes research and quality improvement initiatives.

Deep learning applications in medical imaging have demonstrated capability for automated tasks, yet hernia-specific implementations remain limited. While AI has shown promise in other surgical specialties, the unique challenges of hernia imaging—including variable anatomical presentations, surgical mesh artifacts, and the need for precise boundary delineation—require specialized approaches [7]. This study presents a deep learning system specifically designed for automated ventral hernia assessment, addressing the clinical need for rapid, standardized, and reproducible preoperative analysis.

## Materials and methods

### Data collection

We acquired 215 abdominal CT volumes with ventral hernias. Of the 215 volumes, 85 CTs were used to annotate the xiphoid process and pubic symphysis, another 85 CTs for annotating the hernia defect and hernia sac, and the remaining 45 CTs for annotating the abdominal cavity region, rectus muscles, and psoas muscles. In addition to these annotations, we also acquired 85 abdominal CT volumes from the VerSe20 dataset, which includes labeled vertebrae annotations. All the scans used in the study were supine position breathhold CT acquisitions of the abdomen and pelvis without intravenous (IV) contrast in multidetector 8- to 64-slice CT scanners. To ensure proper segmentation of a diverse range of CT images, the algorithm was designed to accommodate a wide range of tube currents and slice thicknesses, producing significant variations. Data was sourced after Internal Review Board approvals from two centers: Madras Medical College, Chennai, India, and Grant Medical College, Mumbai, India. The study was Health Insurance Portability and Accountability Act of 1996 (HIPAA) compliant and adhered to protocols set by National Ethical Guidelines for Biomedical and Health Research involving Human Participants, Indian Council for Medical Research (ICMR) [8]. All CT volumes were fully de-identified prior to transfer, with patient identifiers removed from DICOM metadata at the source institution in accordance with HIPAA requirements. The use of separate CT subsets for different structure annotations was a deliberate design choice: anatomical structures were annotated in the imaging plane that provided optimal visibility (sagittal for xiphoid/pubic symphysis landmarks, coronal for vertebral structures, axial for hernia defect, sac, and abdominal wall musculature). Annotating all structures simultaneously on every scan would have introduced annotation fatigue and reduced labeling consistency, a recognized risk in large-scale medical image labeling projects. This multi-task annotation strategy, while non-standard in some conventional computer vision benchmarks, is established practice in multi-structure medical imaging pipelines where structures are best delineated across different planes and imaging contexts. Table 1 summarizes the dataset allocation, imaging plane, and annotation details for each model in the HIA system.

**TABLE 1:** Dataset allocation, imaging plane, and annotation summary per HIA model.

Model/Structure	N Cases	Imaging Plane	Approx. Slices/Scan	Source Dataset
Xiphoid & pubic symphysis (landmarks)	85	Sagittal	1–2	Internal (2 centres)
Hernia defect & sac segmentation	85	Axial	1–3	Internal (2 centres)
Abdominal cavity, rectus, psoas segmentation	45	Axial	1–3	Internal (2 centres)
Vertebral landmark (L3/VerSe20)	85	Coronal	1–2	VerSe20 (public)
Abdominal region detection	215	Axial	Pipeline	All above (combined)

Subsets are non-overlapping by design. Mesh was annotated on the same 85-CT defect/sac subset (axial plane, 10–20 slices/scan). N = number of CT volumes.

### Inclusion and exclusion criteria

Scans of patients over the age of 18, undergoing elective ventral hernia repair were included in the study. All kinds of ventral hernia like umbilical, epigastric, incisional, lateral were included. Those images with artifacts were excluded. Randomization was done to ensure inclusion of a wide range of ventral hernia types and sizes in both males and females [9].

### Image annotation

Ground truth was created by manual annotation of the hernia sac, and defect. For proper localisation of the defect, landmarks such as xiphoid, pubic symphysis, and rectus abdominis were also marked. Annotation was done by two radiologists each with more than 5 years of experience in abdomen imaging. Ground truth segmentation maps were created using open-source ITKSNAP ver3.8 software [10]. Each radiologist independently annotated a representative subset of cases for each structure type. Annotations were then cross-reviewed: where discrepancies exceeded a predetermined threshold, the case was discussed and adjudicated by consensus between both annotators. The final consensus segmentation maps were used as ground truth for all model training and evaluation. Sagittal slices were annotated for xiphoid process and pubic symphysis localization; coronal slices were annotated for vertebral landmarks from the VerSe20 dataset; and 512 × 512 axial slices were annotated for hernia defect, sac, abdominal cavity, rectus muscles, and psoas muscles. The number of annotated slices per volume varied by anatomy, with approximately 1–3 representative slices annotated per structure per scan at the relevant anatomical level. This sparse annotation strategy was adopted to balance annotation workload with the need for diverse case coverage across the 215-scan dataset. Each model performs inference on the full input volume at the relevant imaging plane; the sparse annotations provided landmark-level supervision from which the models generalise across adjacent slices. This approach is consistent with established practices in weakly-supervised and semi-supervised medical image segmentation, particularly in settings where dense volumetric annotation is resource-prohibitive. The implications of this strategy for whole-volume segmentation robustness are discussed in Section *Image pre- and post-processing*. Formal inter-rater agreement statistics (e.g., Cohen’s kappa, intraclass correlation coefficient) were not prospectively computed in this study; this is acknowledged as a limitation in Section *Image pre- and post-processing*. Surgical mesh annotation was performed on the same subset of 85 CT scans used for hernia defect and sac annotation, ensuring consistency between defect and mesh labels and enabling the model to learn their spatial relationship directly. Annotations were performed primarily on axial CT slices, which provide the most consistent visualisation of mesh structure and its relationship with surrounding anatomy; coronal and sagittal reconstructions were used as supplementary references where necessary to confirm anatomical consistency. On average, 10–20 axial slices per scan were annotated for mesh, depending on the extent and visibility of mesh within each volume. Mesh annotation presented specific challenges not encountered with soft-tissue structures: low contrast between mesh material and surrounding soft tissue made precise boundary delineation difficult in certain regions, and proximity to the hernia defect meant that overlapping anatomical structures sometimes obscured mesh edges. These challenges were mitigated by careful cross-referencing across imaging planes and slice-by-slice consistency checks during the consensus review process.

### Image pre- and post-processing

We applied a HU window with a range of −200 to 200 to all the CT volumes. We then extracted sagittal slices from the volumes corresponding to the xiphoid process and pubic symphysis annotations, resizing these slices to 128 × 128 and applying min-max normalization. Similarly, we extracted coronal slices from the volumes corresponding to vertebrae annotations, resized them to 128 × 128, and applied min-max normalization. Additionally, we extracted 512 × 512 axial slices from the volumes corresponding to hernia defect, sac, abdominal cavity region, and rectus muscles annotations, and applied min-max normalization to these slices as well.

### Deep learning architecture

**FIGURE 1:**
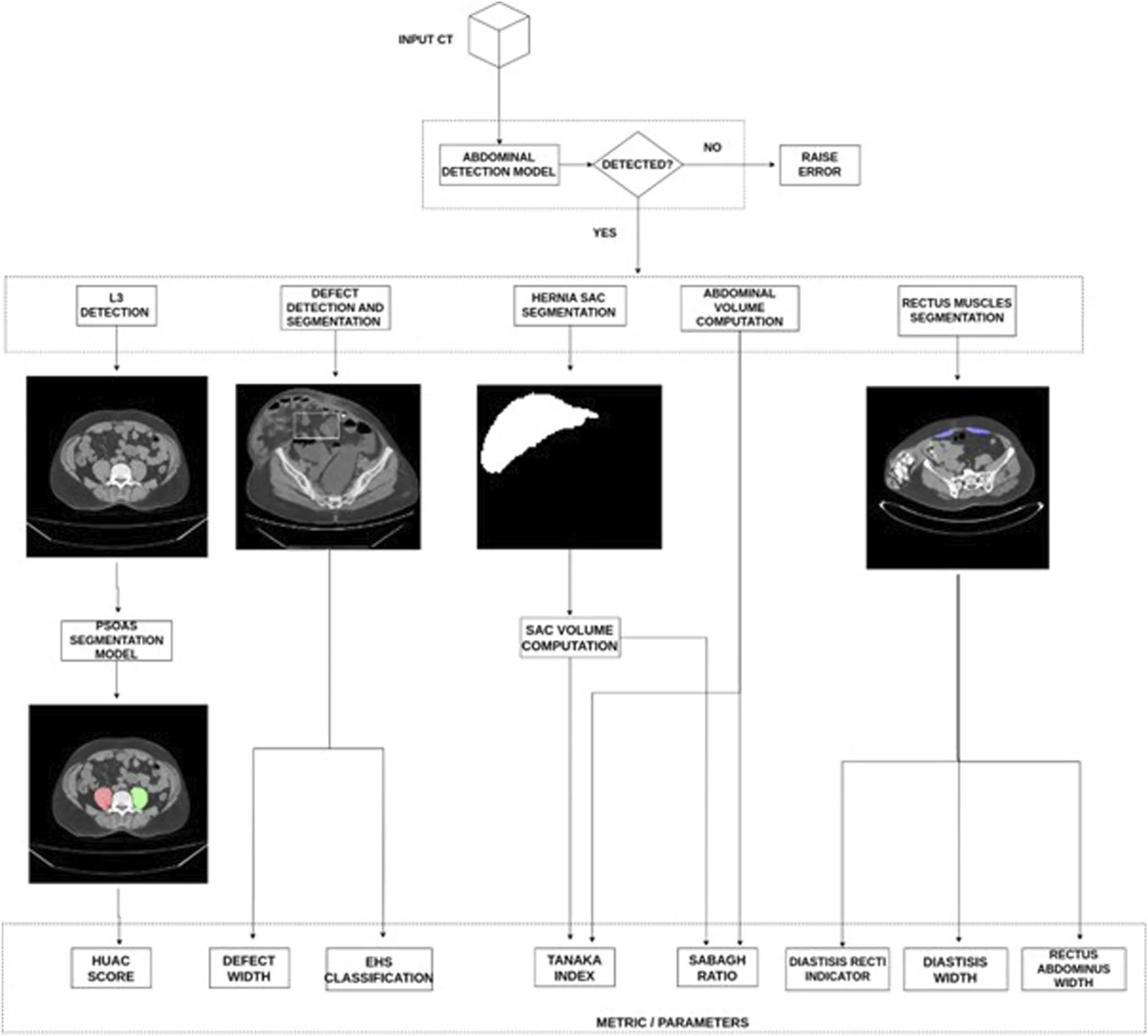
Hernia Image Assist system that includes several DLMs to produce metrics/parameters.

The backend algorithm of the HIA (Hernia Image Assist) system involves two initial tasks. First, it preprocesses the input CT volume to prepare it for further analysis. This preprocessing includes the use of a Deep Learning model (DLM) to identify the abdominal region. Once the presence of the abdominal region is ascertained in the input CT volume, the system advances to subsequent analysis stages, utilizing multiple Deep Learning models (DLMs) and computer vision algorithms. These stages generate a variety of metrics, including but not limited to the defect width, EHS (European Hernia Society) classification (for defect localization), Tanaka index, and volumetric ratios as shown in Figure 1. It should be noted that these downstream clinical metrics are derived outputs of the segmentation pipeline. While their computation is demonstrated in Figure 1, formal validation of their accuracy against clinical measurements was not performed in this proof-of-concept study and is a defined objective of future prospective work. The models for L3 detection and psoas detection were developed to measure the Hounsfield Unit Attenuation Coefficient to predict sarcopenia [11].

**FIGURE 2:**
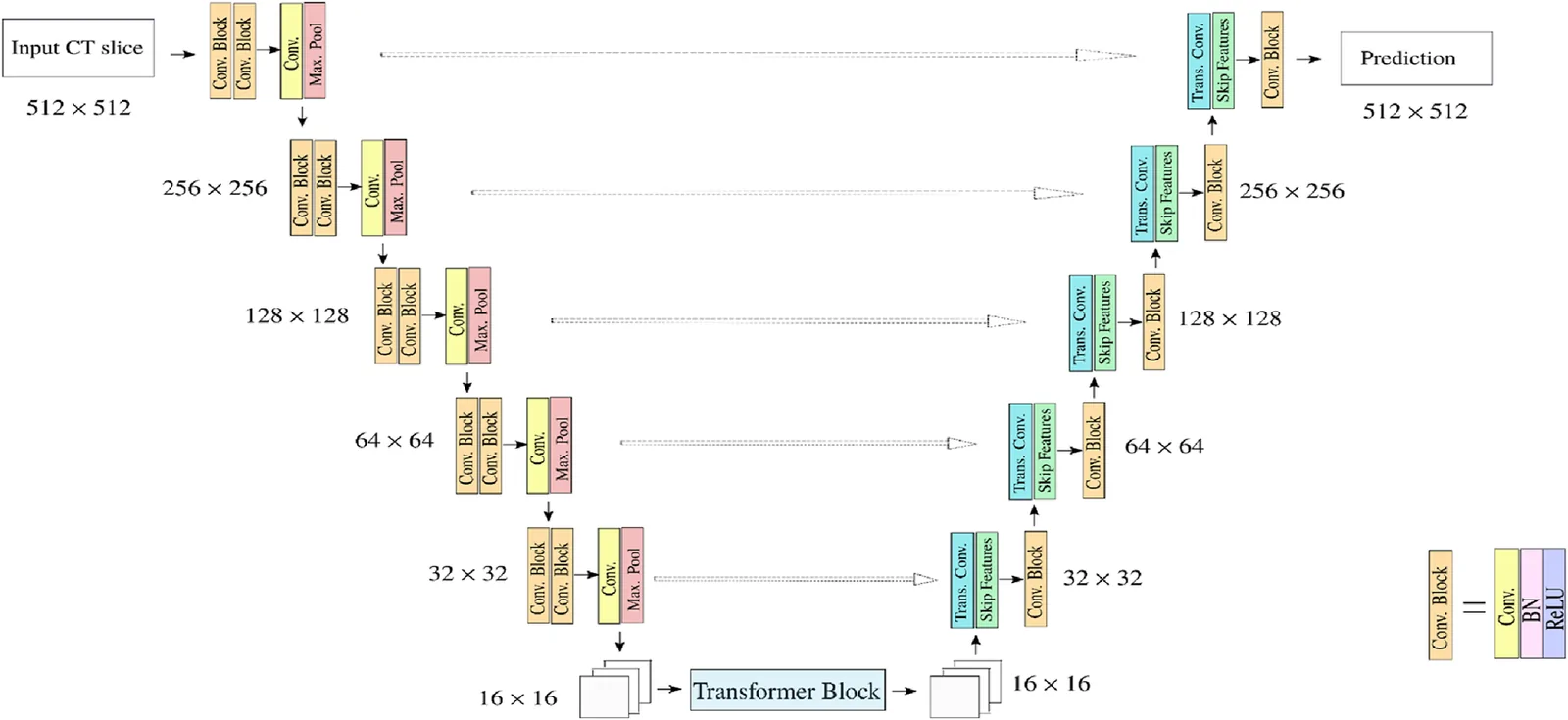
Architecture inspired by TransUNet used to train different DLM of HIA.

To train the multiple deep learning models (DLMs) used in our proposed HIA system, we adopted a common architecture inspired by TransUNet, as shown in Figure 2.

For the DLMs that utilize 128 × 128 size input, we modified the architecture illustrated in Figure 2, by removing two encoder and decoder layers. The training details for each model are provided in the following section.

### Model training and optimization

**FIGURE 3:**
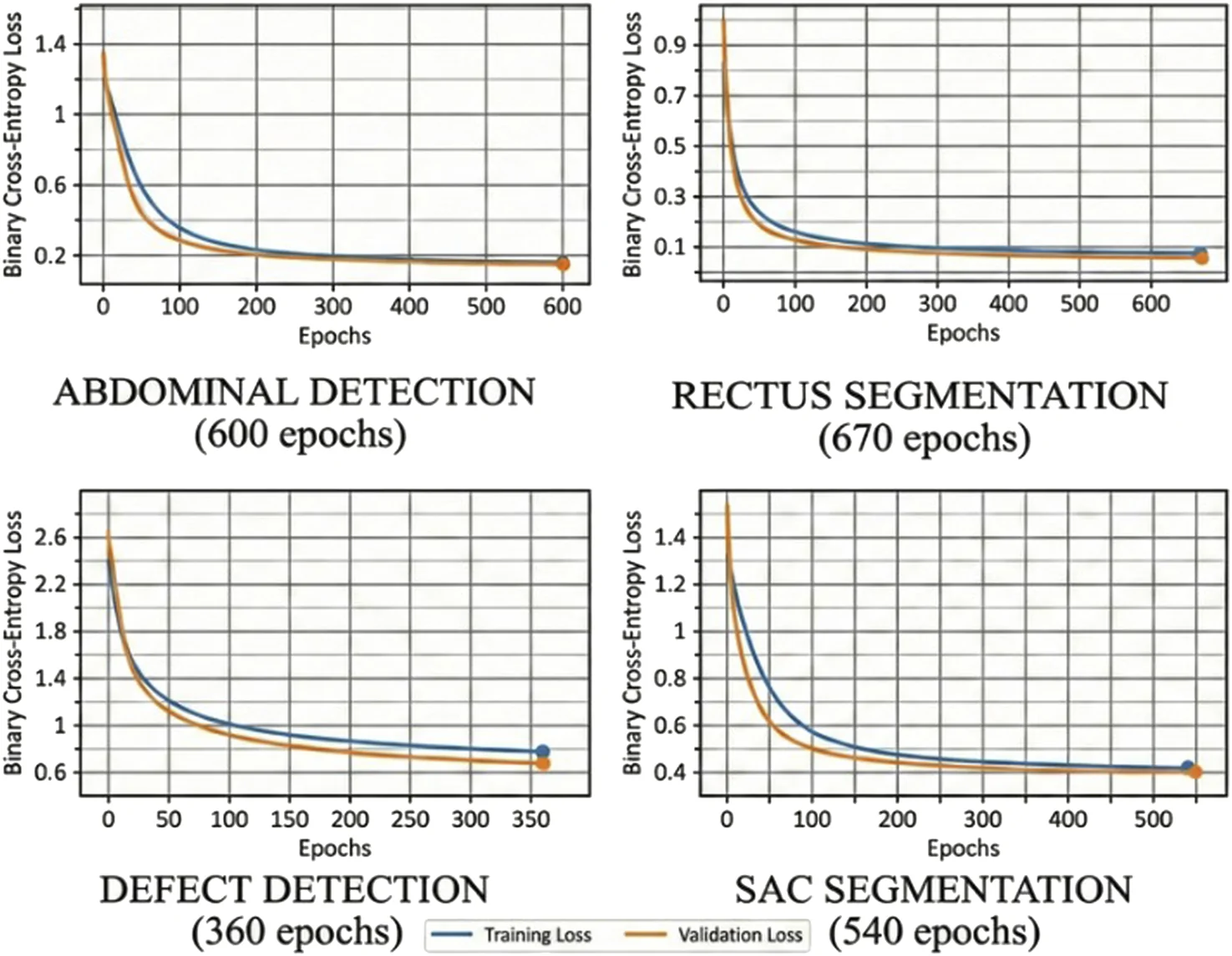
Training and validation loss curves for each deep learning model (DLM) in the HIA system: (top-left) Abdominal Detection model (600 epochs); (top-right) Rectus Segmentation model (670 epochs); (bottom-left) Defect Segmentation model (360 epochs); (bottom-right) Sac Segmentation model (540 epochs). Blue lines indicate training loss and orange lines indicate validation loss. Parallel trajectories indicate minimal overfitting across all models.

The model was trained on different datasets for a total number of epochs as shown in Figure 3. The data was split into 80% for training and 20% for validation. No independent external test set was employed in this proof-of-concept study; this limitation is acknowledged in Section *Image pre- and post-processing*.

For training purposes, we used the TensorFlow deep learning framework on an RTX 4090 24 GB GPU, with a batch size of 10. Each model was optimized using the Adam optimizer and binary cross-entropy loss function. In all cases, patches of size 4 × 4 were processed by the vision transformer. The models were trained for different numbers of epochs depending on the task: abdominal detection was trained for 600 epochs, defect segmentation for 360 epochs, sac segmentation for 540 epochs, rectus segmentation for 670 epochs. The train val curves of different DLMs of HIA are as follows:

## Results

The training and validation curves of different deep learning models of the algorithm are shown in Figure 3. All models showed progressive improvement in performance metrics during training with minimal overfitting, as evidenced by the parallel trajectories of training and validation loss curves.

**FIGURE 4:**
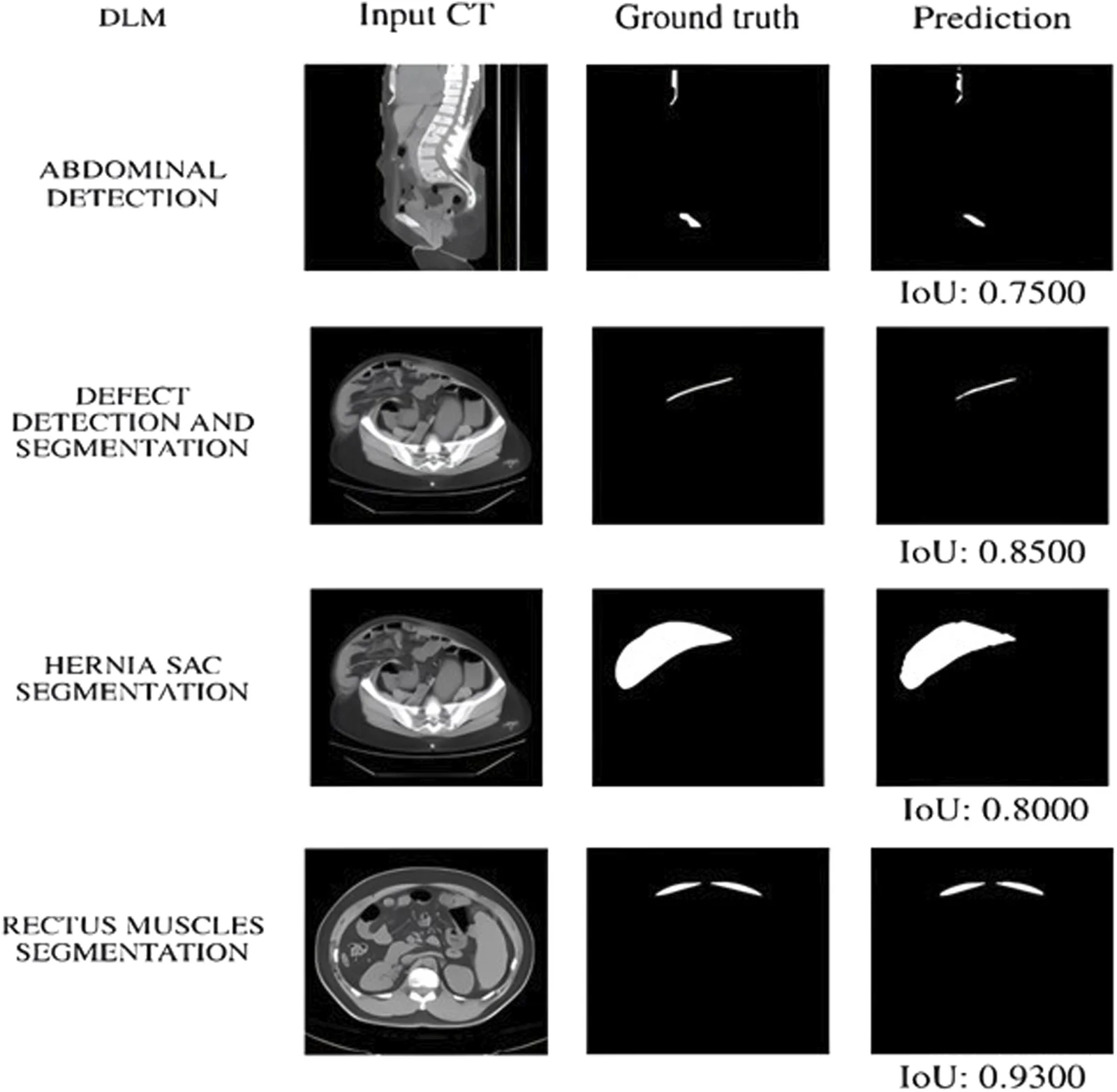
Input CTs, ground truth and model predictions for various anatomical elements.

The input CTs, ground truth and the model predictions for various elements are shown in Figure 4. The automated segmentation achieved precise delineation of critical structures including hernia defects, hernia sacs, and abdominal wall musculature.

The models achieved robust performance across different anatomical structures with high Intersection over Union (IoU) values. For hernia defect, the IoU value was approximately 0.85, while hernia sac segmentation achieved approximately 0.82. Rectus abdominis muscles were segmented with an IoU of approximately 0.89, lateral abdominal wall muscles with 0.87, and psoas muscles with 0.91. These IoU values correspond to Dice Similarity Coefficients (DSC = 2·IoU/(1 + IoU)) of approximately 0.92 for hernia defect, 0.90 for hernia sac, 0.94 for rectus abdominis, 0.93 for lateral abdominal wall muscles, and 0.95 for psoas muscles. Table 2 summarizes the complete performance metrics. It should be noted that IoU and DSC measure spatial overlap with expert annotations and do not directly validate the clinical accuracy of derived measurements such as defect width or volumetric ratios; dedicated validation studies comparing automated measurements against intraoperative findings are required to establish clinical measurement accuracy.

**TABLE 2:** Segmentation performance per anatomical structure.

Anatomical Structure	IoU	DSC
Hernia defect	0.85	0.92
Hernia sac	0.82	0.90
Rectus abdominis muscles	0.89	0.94
Lateral abdominal wall muscles	0.87	0.93
Psoas muscles	0.91	0.95

IoU, intersection over union; DSC, Dice Similarity Coefficient. DSC, was derived from IoU as 2 × IoU/(1 + IoU). Values are approximate and were obtained on the held-out validation split.

## Discussion

This study presents an automated deep learning system for ventral hernia assessment that achieved IoU values ranging from 0.82 to 0.91 across different anatomical structures. These findings contribute to the growing body of literature examining automated approaches to hernia evaluation, where manual assessment variability remains a persistent challenge affecting clinical decision-making in up to 56% of cases [5]. The inter-observer variability in current practice, with intraclass correlation coefficients ranging from 0.21 to 0.737 across different measurement modalities, highlights the need for standardized assessment methods [5, 12].

Current manual hernia assessment faces inherent challenges that affect both clinical care and research validity. Studies report disagreement between radiologists and surgeons on CT scan interpretations in 73% of cases, with experienced abdominal wall reconstruction surgeons correctly identifying previous repair types in fewer than 50% of cases [13]. These inconsistencies arise from the complexity of hernia assessment, which requires evaluation of multiple parameters including defect dimensions, anatomical landmarks, muscle integrity, and volumetric relationships [14]. The manual annotation process in this study required 25–30 min per case, reflecting the time-intensive nature of comprehensive hernia evaluation. Automated systems process these same parameters within a couple of minutes, though the clinical significance of this time reduction requires further investigation in real-world surgical workflows.

### Comparison with existing systems

This study represents one of the first applications of transformer-based deep learning specifically to CT segmentation of ventral hernia anatomy. Earlier automated segmentation work by Xu et al. [15] demonstrated the feasibility of abdominal wall delineation in ventral hernia CT using level-set methods in four cases, achieving mean surface errors under 2 mm for the outer abdominal wall. Tustison et al. [16] extended this to 20 cases using texture-augmented level-set segmentation. The present work advances substantially beyond these foundational studies by deploying multi-structure deep learning segmentation across 215 cases and achieving IoU values of 0.82–0.91 across five anatomical structures. For context in the broader abdominal CT segmentation literature, deep learning models for muscle segmentation at the L3 vertebral level report Dice scores of 0.80–0.93 for major muscle groups, consistent with our findings (see Table 2). Direct comparison with these benchmarks is limited by differences in task definition and dataset characteristics, and head-to-head evaluation under standardized conditions is recommended for future work. Several AI systems for hernia outcome prediction have also been reported. The Atrium Health neural network reports 75% accuracy for predicting component separation requirements and 90% accuracy for wound infection prediction [17]. Machine learning models from other institutions demonstrate 84%–85% accuracy for recurrence and readmission prediction, though these systems focus primarily on outcome prediction rather than anatomical segmentation [18]. These comparisons are contextual only, as different endpoints and training datasets preclude direct performance benchmarking.

The Elhage group’s convolutional neural network, trained on 9,303 CT images from 369 patients, achieved 81.3% accuracy in surgical complexity prediction with an AUC of 0.898 for surgical site infection prediction [19]. These systems utilize various architectures including standard CNNs, U-Net variants, and Feature Pyramid Networks, each with specific advantages and limitations [20]. The TransUNet-inspired architecture employed in our study represents one approach among many, with comparative effectiveness requiring direct head-to-head evaluation under standardized conditions.

Current commercial platforms focus primarily on 3D visualization rather than automated measurement, converting 2D imaging to 3D models for surgical planning. The integration of automated segmentation with these visualization tools remains an area of active development across multiple vendors and research groups.

### Clinical context and standardization

Studies from specialized hernia centers demonstrate associations between standardized protocols and clinical outcomes, including reduced recurrence rates and shorter hospital stays [21]. German hernia center data show reoperation rates decreasing from 8.24% to 3.66% for incisional hernias following standardization initiatives, though multiple factors beyond assessment standardization likely contribute to these improvements [22]. These observations suggest potential benefits from consistent evaluation methods, though causality remains difficult to establish definitively.

Measurement variability affects clinical decisions including mesh selection and surgical approach in approximately half of cases, based on survey data from practicing surgeons [5]. This variability also complicates research efforts, as outcome studies rely on consistent baseline measurements for valid comparisons. Automated systems offer consistent measurements by design, though whether this consistency translates to improved clinical outcomes requires prospective validation. Volumetric CT analysis, including hernia sac and abdominal cavity volume calculation, has been proposed as a comprehensive approach to hernia assessment in complex cases [23]. In our study, we have attempted the same.

Computer vision applications in other surgical domains provide relevant context, with laparoscopic phase recognition systems achieving 94% accuracy and automated tumor segmentation reducing processing time from 5 to 80 min to under 4 min [24, 25]. These parallel developments suggest potential applicability to hernia surgery, though direct extrapolation should be approached cautiously given domain-specific challenges.

### Implementation considerations

AI adoption in surgery faces multiple barriers that affect real-world implementation. Survey data indicate acceptance rates vary significantly by surgeon demographics, with younger surgeons showing greater openness to AI-assisted tools [26]. Concerns about algorithmic transparency, liability, and workflow disruption represent common themes across multiple studies examining healthcare AI adoption [27].

Successful implementation examples emphasize iterative development, user engagement, and integration with existing workflows [28]. Technical challenges include ensuring algorithm generalizability across different imaging protocols, managing data privacy concerns, and establishing appropriate validation frameworks. Federated learning approaches offer potential solutions for multi-institutional collaboration while maintaining data security, though practical implementation remains limited [29]. Infrastructure requirements, including computational resources and integration with existing PACS and EMR systems, represent additional considerations for clinical deployment [30].

### Limitations and clinical considerations

Several important limitations of this study require acknowledgment. First, formal inter-rater agreement statistics between the two annotating radiologists were not prospectively computed. While a consensus review process was employed, future studies should report Cohen’s kappa or intraclass correlation coefficients to quantify annotation reliability. Second, performance metrics beyond IoU and DSC (including Precision, Recall, F1-score, and mAP50) were not reported in this study. These metrics would provide a more complete characterization of model behavior, particularly regarding false positive rates. Third, the absence of an independent external test set means that all reported IoU values were derived from a held-out validation split of the same institutional dataset, limiting the strength of generalizability claims. Fourth, no subgroup analysis by hernia type (umbilical, incisional, epigastric, lateral) or defect size was performed; future studies should stratify performance metrics accordingly. Fifth, the split-dataset annotation strategy, while pragmatically motivated, may introduce heterogeneity in ground truth quality across structure types. The study’s focus on clear anatomical presentations may not fully represent the complexity encountered in multiple recurrent hernias or cases with extensive surgical mesh. Dataset size and diversity represent ongoing challenges for AI model development in hernia surgery. The specialized nature of complex hernia presentations requires larger, more diverse training datasets to ensure algorithm generalizability across different patient populations and institutional settings [31].

The current system’s reliance on non-contrast CT imaging, while practical for routine clinical use, may limit assessment accuracy in cases requiring detailed soft tissue differentiation or vascular anatomy evaluation. Future iterations should consider incorporating contrast-enhanced imaging protocols when clinically indicated [32]. Additionally, the absence of dynamic imaging assessment limits evaluation of hernia reducibility and fascial compliance, factors that influence surgical approach decisions [33].

The training dataset’s geographic limitation to two Indian institutions limits generalizability to other populations and healthcare settings. Hernia characteristics, body habitus, and imaging protocols vary across regions, potentially affecting algorithm performance. External validation across diverse populations remains essential before clinical deployment. Detailed patient-level cohort characteristics including age, sex, BMI, hernia subtype distribution, recurrence status, and prior mesh repair history were not available for tabulation at this stage of the study. The absence of a formal cohort characteristics table is acknowledged as a reporting limitation; future prospective studies should collect and report these variables systematically to allow appropriate subgroup performance analysis.

### Future research directions

Based on the specific findings and limitations of the present study, several targeted future research directions can be identified. First, and most critically, external validation of the HIA segmentation models on datasets from geographically diverse institutions is required before any clinical deployment. Second, prospective studies comparing automated defect measurements (width, area, volumetric ratios) against intraoperative findings or surgical pathology would establish whether the technical accuracy demonstrated here translates into clinically meaningful measurement precision. Third, performance should be evaluated across hernia subtypes and defect size categories; the current dataset was not sufficiently powered for such subgroup analyses. Fourth, extension to cases involving prior mesh repair, which represent the most challenging and clinically consequential presentations, is an important next step. Fifth, the annotation methodology should be formalized with prospective inter-rater agreement quantification. The broader research landscape in AI-assisted hernia surgery also highlights multi-institutional validation and cost-effectiveness analyses as priorities [34], and a recent scoping review identified only 20 relevant articles in this area, indicating significant opportunity for further investigation [35].

Potential applications extend beyond preoperative assessment to include intraoperative guidance and postoperative monitoring. Augmented reality navigation, automated mesh positioning, and AI-enhanced training simulators represent areas of active development [36, 37]. Integration with established clinical guidelines from hernia societies could standardize implementation approaches, though optimal integration strategies remain undefined [38].

Development of interpretable AI systems that provide clear rationales for their outputs represents an important consideration for clinical acceptance. Multi-institutional collaboration for dataset development and validation will be necessary to ensure broad applicability [39]. Prospective clinical trials comparing automated versus manual assessment on patient outcomes remain absent from the literature and should be prioritized.

### Conclusion

This study demonstrates the technical feasibility of automated ventral hernia assessment using deep learning methods, achieving segmentation IoU values of 0.82-0.91 (DSC 0.90-0.95) across five anatomical structures, representing a proof-of-concept advance over earlier non-deep-learning segmentation approaches in this domain. These results indicate reasonable agreement with expert radiologist annotations under the study conditions. The system offers potential advantages in consistency and processing efficiency; however, the current work is limited to a two-center Indian dataset, uses IoU-based technical metrics only, and has not been externally validated or compared with intraoperative measurement standards. Translation to clinical utility requires rigorous prospective validation studies examining measurement accuracy, impact on surgical decision-making, and patient outcomes. These limitations notwithstanding, this work establishes a foundation for further development of automated hernia assessment tools and identifies specific priorities for the next phase of investigation.

## Data Availability

The raw data supporting the conclusions of this article will be made available by the authors, without undue reservation.
